# Two waves of anisotropic growth generate enlarged follicles in the spiny mouse

**DOI:** 10.1186/2041-9139-5-33

**Published:** 2014-09-25

**Authors:** Sophie A Montandon, Athanasia C Tzika, António F Martins, Bastien Chopard, Michel C Milinkovitch

**Affiliations:** 1 Department of Genetics & Evolution, Laboratory of Artificial & Natural Evolution (LANE), University of Geneva, Sciences III, 30, Quai Ernest-Ansermet, Geneva 4 1211, Switzerland; 2 Department of Computer Science, Scientific and Parallel Computing Group, University of Geneva, Geneva, Switzerland

**Keywords:** *Acomys*, Evolutionary developmental biology, Hair, Spines, Spiny mouse

## Abstract

**Background:**

Mammals exhibit a remarkable variety of phenotypes and comparative studies using novel model species are needed to uncover the evolutionary developmental mechanisms generating this diversity. Here, we undertake a developmental biology and numerical modeling approach to investigate the development of skin appendages in the spiny mouse, *Acomys dimidiatus*.

**Results:**

We demonstrate that *Acomys* spines, possibly involved in display and protection, are enlarged awl hairs with a concave morphology. The *Acomys* spines originate from enlarged placodes that are characterized by a rapid downwards growth which results in voluminous follicles. The dermal condensation (dermal papilla) at the core of the follicle is very large and exhibits a curved geometry. Given its off-centered position, the dermal papilla generates two waves of anisotropic proliferation, first of the posterior matrix, then of the anterior inner root sheath (IRS). Higher in the follicle, the posterior and anterior cortex cross-section areas substantially decrease due to cortex cell elongation and accumulation of keratin intermediate filaments. Milder keratinization in the medulla gives rise to a foamy material that eventually collapses under the combined compression of the anterior IRS and elongation of the cortex cells. Simulations, using linear elasticity theory and the finite-element method, indicate that these processes are sufficient to replicate the time evolution of the *Acomys* spine layers and the final shape of the emerging spine shaft.

**Conclusions:**

Our analyses reveal how hair follicle morphogenesis has been altered during the evolution of the *Acomys* lineage, resulting in a shift from ancestral awl follicles to enlarged asymmetrical spines. This study contributes to a better understanding of the evolutionary developmental mechanisms that generated the great diversity of skin appendage phenotypes observed in mammals.

## Background

Although hair is one of the defining features of the Class Mammalia and is found in most of its species, mammalian skin appendages can take a diversity of other phenotypes such as the scales of pangolins, the scutes of armadillos, or the spines of echidnas and hedgehogs. So far, these spectacular morphologies have not been reproduced in transgenic mice, although there exist several strains with modified hair coat, such as *Fgf5* mutants with longer hair [1], hairless mice (see [2] for a review), or multiple lines with coarse hair [3-5]. Clearly, understanding the evolution of the diversity of mammalian skin appendages will necessitate an input from the ‘wildlife’ and the introduction of novel model species [6]. To that end, we use, herein, developmental biology and numerical modeling techniques to investigate the mechanisms responsible for spine development in the spiny mouse, *Acomys dimidiatus*.

**Figure 1:**
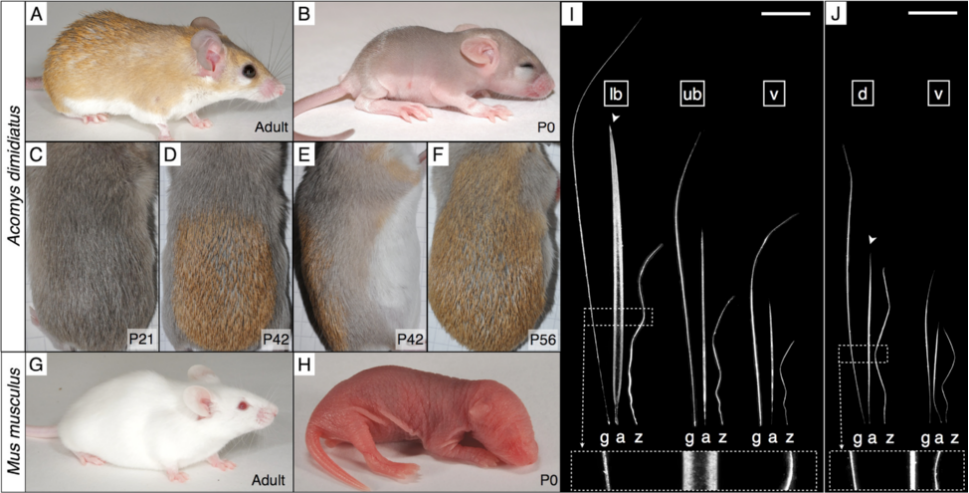
***Acomys *spines versus laboratory mouse hairs*. *(A)** Adult *Acomys* with orange spines on the lower back. **(B)** Newborn *Acomys* with grey coat. **(C)** Dorsal view of a 3-week-old *Acomys*. **(D**, **E)** Dorsal and lateral views of a 6-week-old *Acomys.***(F)** Dorsal view of an 8-week-old *Acomys.***(G)** Adult and **(H)** newborn laboratory mouse (NMRI). **(I)***Acomys* hair types from the lower back (lb), upper back (ub), and ventral side (v). **(J)** Laboratory mouse hair types from the dorsal (d) and ventral (v) sides. Insets: same scale magnification of the images in the dotted frames. g, guard; a, awl; z, zigzag; arrowhead, dorsal awl hairs. Scale bars: 2 mm.

*Acomys* is separated by 25 million years of evolution from the laboratory mouse [7], and derives its common name from the presence of spines, particularly on its lower back (Figure 1A). There is an increasing scientific interest in this murid genus because of its recently-described spectacular regeneration abilities [8], and also its relevance to the study of diabetes, obesity, and reproductive physiology in humans [9,10]. Data is available on the reproduction of *Acomys*[11] and a superovulation protocol has been proposed [12], but little is known of its embryonic development, in particular that of the skin and its appendages. Gestation lasts approximately 38 days (vs. 21 for the laboratory mouse) and the mean litter size is only about 2.5 [13]. *Acomys* newborns have their eyes open and stiff developing hair all over their body (Figure 1B), contrary to the ‘naked’ mouse babies (Figure 1H). Starting at 30 days postpartum (P30), the grey juvenile pelage (Figure 1C) is gradually replaced by the adult coat, made of very coarse (spine-like) hairs with orange tips, that appear as a patch in the middle of the lower back (Figure 1D,E) before spreading over the dorsum (Figure 1F). The process is completed approximately at P60, and coincides with the sexual maturity of the animal [14]. The function of spines in *Acomys* is unknown but is likely involved in avoidance of both predators and aggressive conspecifics. Indeed, in addition to spiny mice exhibiting a cleavage plane between the tail skin and the underlying muscles and vertebrae (greatly facilitating tail skin loss [15]), our observations indicate that *Acomys* individuals escape aggression during interspecific interactions thanks to the easy loss of spines: typically, the aggressor is left with a tuft of spines in its mouth while the aggressed individual escapes. In addition, the volume of the spines makes the animal look bigger.

Mice exhibit four types of hairs with different functions and morphologies [16]: guard, awl, auchene, and zigzag. Using immunostaining, tridimensional (3D) reconstruction, scanning electron microscopy, numerical simulations, and comparisons with laboratory mouse hair development, we show that *Acomys* evolved spines as greatly enlarged awl hairs with a concave morphology. Our analyses indicate that an enlarged and asymmetrical dermal papilla causes two waves of anisotropic growth in the follicle, namely the proliferation of matrix cells at the posterior side and the enlargement of the inner root sheath at the anterior side. The size and shape of the dermal papilla is therefore likely to constitute the initial and primary determinant of the final keratinized form of the spine.

## Methods

### Animals

A colony of captive-bred *Acomys dimidiatus* was established at the LANE, where all experiments were performed in accordance with the Swiss animal welfare and ethical regulations (permit number 1008/3421/0). The NMRI strain of the laboratory mouse was used as reference (Janvier Labs, Le Genest-Saint-Isle, France).

### Hair sampling

Hairs were removed from anesthetized animals from three different regions: the upper back (between the shoulder blades), the middle of the lower back, and the ventral side. Pictures of the sorted hairs were taken with a M80 stereoscope (Leica, Solms, Germany). Gold-coated hairs were visualized with a JEOL-6510LV scanning electron microscope.

### Histology

Tissue samples were fixed overnight in 4% PFA before embedding in paraffin; 7-μm sections were stained with Hematoxylin and Eosin and 1% Alcian blue (HEA) in 3% acetic acid (30 min) or processed for immunohistochemistry. De-paraffinized sections were treated for 30 min with Protease XXV (AP-9006-005, Thermo Scientific, Waltham, MA, USA), and blocked with a solution containing 5% goat serum (G9023, Sigma, St. Louis, MO, USA). Sections were incubated with the primary (Laminin Ab-1, RB-082-A; Keratin, Pan Ab-3, MS-744-A; Thermo Scientific) and secondary (Alexa Fluor, A11008, A11004, Life Technologies, Carlsbad, CA, USA) antibodies. Nuclei were stained with Hoechst 33342 (H1399, Life Technologies). Images were acquired with a Pannoramic MIDI Slide scanner (3D HISTECH, Budapest, Hungary) and a LSM 700 confocal microscope (Zeiss, Oberkochen, Germany).

### Cell division analyses: 5-bromo-2’-deoxyuridine (BrdU) and phospho-Histone H3 (pH3) staining

An *Acomys* was injected intraperitoneally with BrdU (2 mg/100 g) and euthanized 1 h later. Dorsal skin was fixed and serial transversal and longitudinal paraffin sections were prepared. The rehydrated sections were incubated for 10 min in boiling citrate buffer. For the BrdU antibody, the sections were also treated with 0.1% trypsin for 5 min. Sections were blocked using 5% goat serum and bovine serum albumin (2 mg/mL) prior to incubation with the primary (BrdU, sc-56258, and pH3, sc-374669, Santa Cruz Biotechnology, Dallas, TX, USA; Keratin 71, 1/500, NBP2-17040, Novus Biologicals, Littleton, CO, USA) and the secondary (Alexa Fluor, A-11006, A-21428, A-11001, Life Technologies) antibodies. Pictures were acquired with a LSM 700 confocal microscope and a Pannoramic MIDI Slide scanner (3D HISTECH). Cells were manually annotated (separately for each staining) and, for BrdU analyses, six areas were manually defined using in-house developed plugins [17] of MeshLab [18] relative to the localization of the dermal papilla (DP): (i and ii) posterior area with and without BrdU^+^ cells, respectively; (iii and iv) anterior area with and without BrdU^+^ cells, respectively; (v) posterior inner root sheath (IRS); and (vi) anterior IRS (Additional file 1: Table S2). Twenty-four serial transverse sections of 7 μm (total 168 μm) were analyzed for two follicles of a 22-day-old *Acomys*, and 41 sections of 7 μm (total 287 μm) were analyzed for two follicles of a 28-day-old *Acomys* (Additional file 1: Table S2). For pH3 analyses, two regions where defined: (i) posterior area and (ii) anterior area (Additional file 1: Figure S7).

### Cell death analyses (TUNEL assay)

Initially, the sections were treated as described above for BrdU staining and then incubated for 90 min at 37°C in TUNEL reaction mix prepared as described by the manufacturer (11767291910, 11767305001, Roche, Basel, Switzerland). Pictures were acquired with a LSM 700 confocal microscope (Zeiss) and cells were manually annotated. Color-coded representations of cell densities were made with in-house developed plugins of MeshLab (Visual Computing Lab; [18]).

### 3D reconstruction of hair follicle

Seven-micron transverse skin sections were stained using Pan-Keratin (KPan) and Laminin antibodies as described above. Images were acquired with a Pannoramic MIDI Slide scanner (3D HISTECH) and one follicle was selected for 3D-reconstruction. All 102 images (covering a total of 714 μm) were rigidly aligned using Fiji’s Linear Stack Alignment with the SIFT plugin [19]. The green (Laminin) and the red (KPan) channels were then split and the alignment was smoothed using the Elastic Alignment tool of StackAligner [20]. The two smoothed channels were finally merged using ImageJ [21], and movies were prepared using Imaris (Bitplane Scientific Software, Zurich, Switzerland).

### Simulations

We used the finite-element method and the equation system of solid linear elasticity (see Supplementary Text and Appendices for details) to describe the characteristics of the IRS, the cortex, and the off-centered medulla, as well as the deformations they are subjected to (Additional file 1: Figure S7): the protrusion of the IRS and the resulting stress applied to the anterior cortex, a vertical strain exerted on the cortex cells as they elongate during keratinization, and the formation of a foam-like structure in the keratinized medulla.

## Results

### *Acomys* spines are modified awl hairs

**Figure 2:**
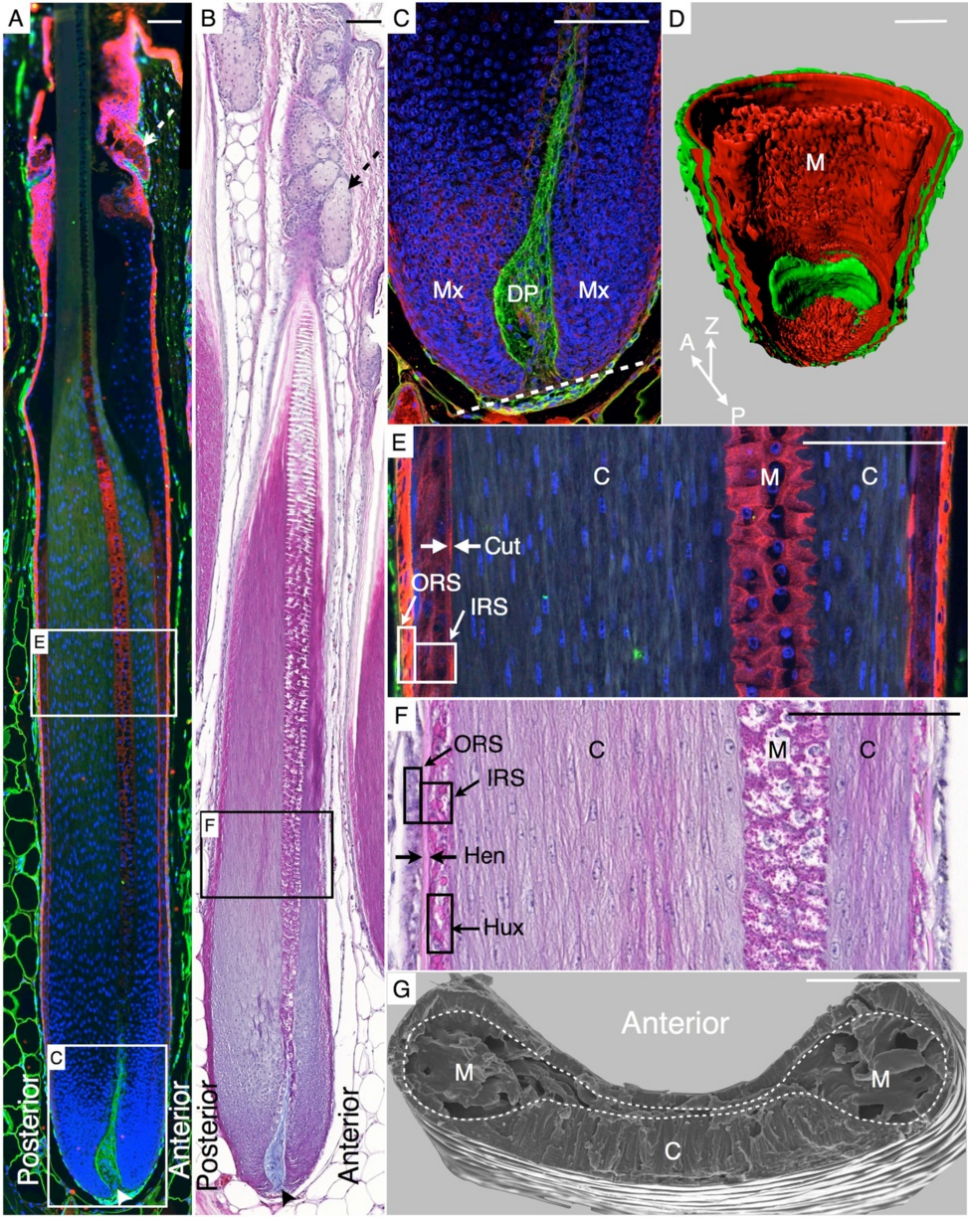
***Acomys *spine morphology at first postnatal anagen phase*. *(A)** Pan-Keratin (red), Laminin (green), and Hoechst (blue) immunostaining and **(B)** HEA coloration of a lower back hair; arrowhead, dermal papilla (DP); dashed arrows, sebaceous gland. **(C)** Detail of A showing the matrix (Mx) and DP*.* The dashed line indicates the tilted base of the follicle. **(D)** 3D-reconstructed follicle from 102 sections (see Supplementary Movie 1). **(E)** Detail of **A**; white frames: outer root sheath (ORS) and inner root sheath (IRS), double facing arrows: IRS cuticle (Cut). **(F)** Detail of **B**; black frames: ORS, IRS, and IRS Huxley’s layer (Hux), double facing arrows: Henle’s layer (Hen). **(G)** Scanning electron micrograph of a lower back adult awl hair fracture. The border between medulla (M) and cortex (C) is delineated with dashed line. Scale bars: 100 μm.

To determine which of the hair types of the mouse coat is evolutionary homologous to *Acomys* spines, we collected hair (Figure 1A-J) from the ventral side as well as the upper and lower back of a subadult *Acomys*, and compared them to the known hair types of the laboratory mouse [16]. Three kinds of hairs were identified in *Acomys* (Figure 1I): (i) guard hairs, which are straight and are the longest of all hair types; (ii) awl hairs, that are the thickest of all hair types, but present a great variation in length and thickness depending on the body part they are found; and (iii) zigzag hairs that are approximately half the length of the guard hairs and are characterized by multiple bends. Although *Acomys* awl hairs on the ventral side and upper back are moderately larger than their mouse counterparts (Figure 1I,J), they gradually become longer and much thicker (hence, spiny) towards the middle of the lower back. Electron microscopy images further reveal the presence of a pronounced furrow at the anterior side of these spines (Figure 2G). Note that awl hairs in the laboratory mouse present a slight but noticeable furrow [22], further supporting their homology with *Acomys* spines. The fourth type of hair, the auchene hair (not shown in Figure 1), is very scarce in the laboratory mouse and we could not identify its homolog in *Acomys*. Based on these observations, we conclude that the *Acomys* spines are modified awl hairs.

### The concentric organization of the *Acomys* spine

Longitudinal paraffin sections of the lower back skin were used for immunostaining with (i) a monoclonal antibody against several keratins (KPan) found labeling specific epidermal layers of the mouse and *Acomys* follicle, and (ii) a polyclonal antibody against Laminin to visualize the DP (Figure 2A,C). This approach, coupled with HEA staining, allowed us to identify all concentric layers that make up the *Acomys* spines (Figure 2A–F) and their correspondence with homologous structures in the laboratory mouse [23] (Additional file 1: Figure S1A–C). Starting from the outermost layer, we observed the outer root sheath (ORS, the continuation of the basal layer of the epidermis) which is only two-cells thick (Figure 2E,F). Adjacent to the ORS is the Henle’s layer (Figure 2F, double-facing arrows), the first of the three layers of the IRS. The thickness of the next layer, Huxley’s layer, is highly variable depending on the side (anterior/posterior) and the height in the follicle, as described below. It is faintly labeled by KPan and contains large trichohyalin granules [24] that render it pink with HEA (Figure 2F). The third IRS layer is the cuticle, made up of a single sheet of small cells that are only visible with the KPan immunostaining (Figure 2E, double-facing arrows).

The core of the follicle is made of the spine shaft, which is the only part that emerges from the skin, and is composed of a hard component, the cortex (‘C’ in Figure 2), and a softer one, the medulla (‘M’ in Figure 2). The HEA staining of the cortex’s elongated cells changes from blue to pink as they keratinize (Figure 2B). In *Acomys*, the cortex is highly asymmetric: it is approximately three to four times thicker at the posterior than at the anterior side of the follicle (Figure 2A-B,E-F). The medulla is made of large round cells that contain small trichohyalin granules and is labeled with KPan.

Below the medulla, we find the DP (arrowhead, Figure 2A–D) which is known in mice to be a key signaling center influencing the follicle formation, growth, and cycling [25]. Its contour is clearly delineated by the Laminin immunostaining and the Alcian blue coloration. The DP is surrounded by a pool of proliferating cells, the matrix (Figure 2C), which will give rise to the IRS, the cortex, and the medulla as they migrate upwards and differentiate. The upper part of the *Acomys* spine follicle is associated with sebaceous glands consisting of large swollen cells (Figure 2A,B, dashed arrows). Homologous layers (epidermis, ORS, IRS, medulla) in the laboratory mouse are shown in Additional file 1: Figure S1B,C.

### The *Acomys* spine is shaped by two waves of anisotropic growth

First, using follicles from a sub-adult *Acomys* in the first postnatal anagen phase, we aligned KPan and Laminin immunostained serial transverse sections to perform 3D reconstruction of the *Acomys* spine follicle. Second, we performed BrdU labeling and pH3 immunostaining of similar serial sections to identify the spatial distribution of cells in the S-phase and M-phase, respectively, in the spine follicle. Third, a Keratin 71 antibody (K71) was used as an immunomarker of the IRS cells and their precursors in the matrix [26]. Finally, the TUNEL assay allowed us to detect apoptosis at different levels of the follicle.

**Figure 3:**
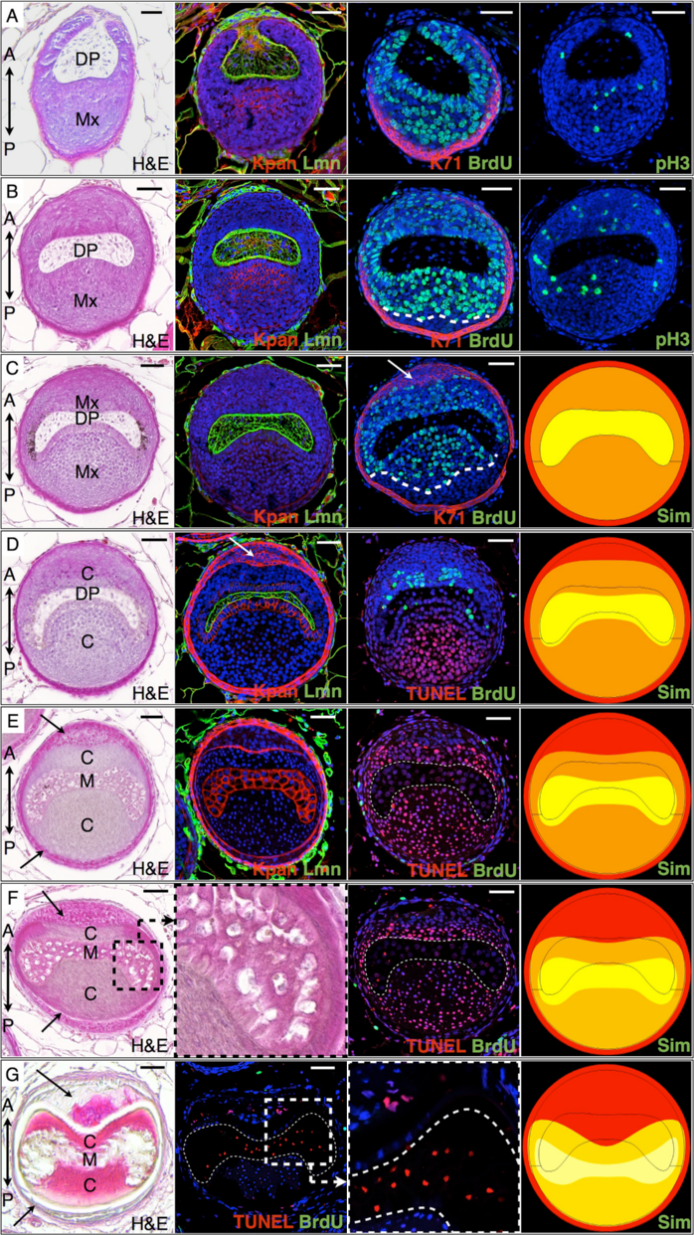
**Observed and simulated cell proliferation and cell death along a spine follicle.** Transverse sections (28-day-old *Acomys*) are taken along the spine long axis, from the basis of the follicle (rows **A-C**), to the DP-medulla transition (rows **D-E**), to the collapse of the keratinized medulla (rows **F-G**). H&E, Hematoxylin and Eosin staining. Immunostaining: Kpan, Pan-Keratin (red); Lmn, Laminin (green); BrdU, 5-bromo-2'-deoxyuridine (green); K71, keratin 71 (red); pH3, phospho-Histone H3 (green); TUNEL (red), Hoechst (blue). Arrows: IRS, DP: dermal papilla, Mx: matrix, C: cortex, M: medulla. Simulated transverse sections (Sim) are snapshots of the Supplementary Movie 2 showing time evolution of a simulated follicle. Scale bars: 50 μm.

**Figure 4:**
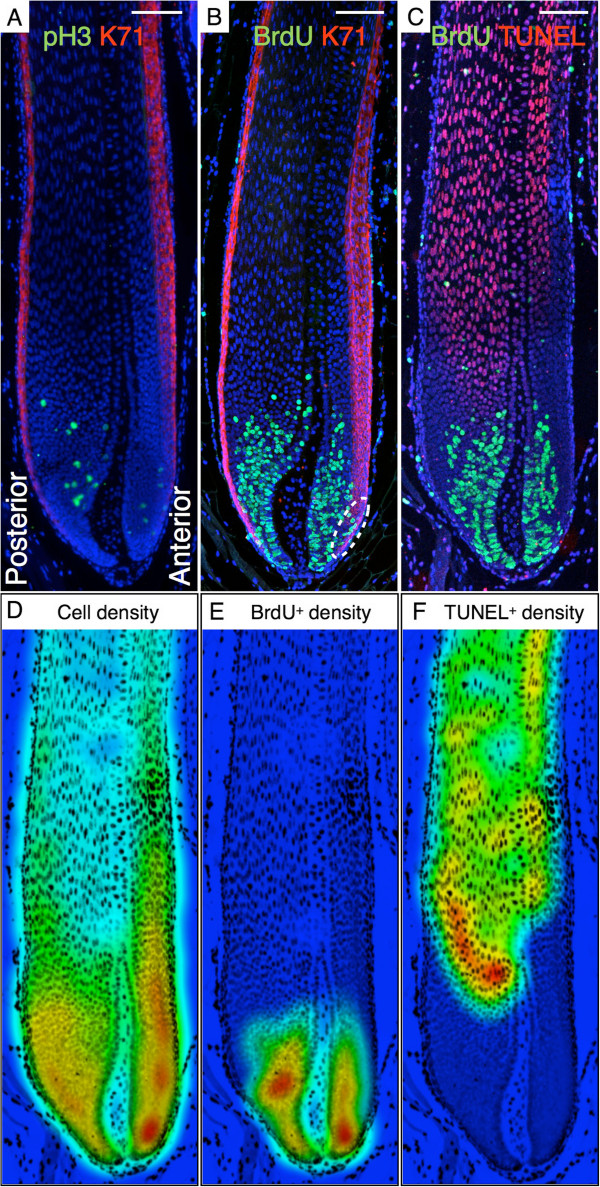
**Densities of proliferating and apoptotic cells in a spine follicle.** Longitudinal sections of a 28-day-old *Acomys.***(A)** pH3 (green), K71 (red), and Hoechst (blue) immunostaining. **(B)** BrdU (green), K71 (red), and Hoechst (blue) immunostaining. **(C)** BrdU (green), TUNEL (red), and Hoechst (blue) immunostaining. **(D–F)** Color-coded local relative densities in a radius that corresponds to 3% of the image diagonal (blue: low density, red: high density) of cells labelled with Hoechst **(D)**, BrdU **(E)**, and TUNEL **(F)**. Scale bars: 100 μm.

The 3D reconstruction of the spine follicle (Additional file 2: Movie S1, Figure 2D) shows that the DP exhibits a complex curved geometry (crescent-shape in transverse section; Figure 3) with two lobes, and the BrdU/pH3/K71/TUNEL labeling (Figures 3,4; Additional file 1: Figures S2–S4,S6,S7) hint at an unexpected mechanism responsible for the asymmetrical development of the *Acomys* spine. The basis of the follicle (dashed line, Figure 2C) is tilted relative to the spine’s long axis. Hence, only the posterior side of the matrix is present in the deepest transverse sections of a spine (Figure 3A, Additional file 1: Figure S2A,S3A). At this level in the follicle, a great number of BrdU^+^ and fewer dividing cells (pH3 stained) are observed (Figure 3A), and the K71 staining labels the posterior IRS precursors, of which some are also proliferating. Higher in the follicle (Figure 3B), the anterior matrix appears and the dermal papilla adopts an oblong curved shape. Consistent with the increase of the transverse-section area of the follicle, a larger number of proliferating cells and total number of cells is observed at both sides of the matrix. Although the number of cells is greater at the posterior side, the cell density is larger at the anterior part (Figure 4D; Additional file 1: Figure S4A,B). In addition, we can observe the anterior IRS precursors, a few of which are BrdU^+^ (oval in Figure 4B).

Moving towards the skin surface along the long axis of the spine (Figure 3C, Additional file 1: Figure S2C,S3C), the DP assumes a crescent shape and the matrix asymmetry becomes more pronounced. It is at this height of the follicle that the posterior matrix exhibits the maximum number of cells, approximately 20% of which maintain BrdU labeling (Additional file 1: Figure S4A). Moving upwards in the follicle, we observe a gradual drop in cell density because the total number of cells remains roughly constant but the transverse-section area continues to increase. The posterior IRS attains its final thickness and then remains invariable throughout the follicle length with no further proliferation.

From this level up, the anterior IRS experiences a substantial enlargement as it forms a protrusion towards the developing cortex (Figure 3C–E). This is associated with a rapid increase of the total cell number within this layer and the continuous presence of BrdU^+^ cells (Additional file 1: Figure S4D). Even higher in the follicle (Figure 3D,E), the DP gets thinner and is surrounded by round cells, which will eventually form the medulla. The latter’s shape and position are similar to those of the underlying DP in the resulting hair shaft. The percentage of proliferating cells gradually decreases as the matrix cells differentiate and form the cortex, with the transition occurring faster at the posterior side (Figure 3D,E and 4C; Additional file 1: Figure S2). At this level (Figure 3E,F), the follicle has acquired its main characteristics: (i) a crescent-shaped medulla, (ii) a posteriorly-enlarged cortex, and (iii) an expanded anterior IRS (the Huxley’s layer, in particular). The anisotropic growths of the cortex and IRS play an important role in generating the final shape of the spine.

### The impact of keratinization on spine morphology

Shortly after the DP-medulla and matrix-cortex transitions, first the cortex cells and then the medulla cells start to keratinize: this is a particular form of apoptosis, characterized among others by chromatin condensation, DNA fragmentation, and accumulation of keratin intermediate filaments (IF) in the cytoplasm. However, the keratinization process differs significantly between the cortex and the medulla. Indeed, cortex cells become vertically elongated and accumulate massive amounts of IF (as evidenced by the HEA staining that changes from blue to pink; Figure 2A), forming a compact layer of keratinized cells in the spine (Additional file 1: Figure S5A). DNA fragmentation starts at the level where cell proliferation ends, as indicated by the transition from BrdU^+^ to TUNEL^+^ cells (Figure 3C–E and 4C). Note that the apoptosis onset (i) starts at a lower level of the follicle in the posterior than in the anterior cortex (Figure 4C) and (ii) anti-correlates with cell density, suggesting that high cell density might inhibit differentiation/keratinization. On the other hand, medulla cells maintain a roundish shape during keratinization (inset of Figure 3F). While the *Acomys* spine cortex gradually forms horizontal keratinized extensions within the medulla space (as observed in mouse hairs [27]), the medulla cells shrink and die (Figure 3F,G), leaving behind empty cavities delimited by a reticulated structure (Additional file 1: Figure S5B). Note that apoptosis in the IRS starts when the process is almost completed in the hair shaft (cortex and medulla) (inset of Figure 3G).

During keratinization (Figure 3D–G; Additional file 1: Table S1), the transverse-section area of the medulla remains relatively stable, that of the anterior IRS increases, while the posterior and anterior cortex areas substantially and rapidly decrease due to cell elongation (parallel to the long axis of the spine) and the accumulation of IF. Given its initially large size, the collapse of the posterior cortex has a particularly dramatic effect on the overall morphology of the spine, i.e., the matrix surface (relative to the whole follicle) drops from 67% of the total transverse-section area at the base of the follicle (Figure 3C,D) to a relative cortex area of 27% at the level of the keratinized spine shaft (Figure 3G; Additional file 1: Table S1). Even higher in the spine, the central part of the medulla collapses, yielding the final transverse-sectional shape of the spine (Figure 2G). Thus, the deep furrow observed in the emerging spine shaft results from two major events: (i) the protrusion of the anterior IRS within the matrix/cortex and, (ii) much higher in the follicle, the keratinization process that results in a remarkable decrease of the cortex transverse-section area. This complex process involving two waves of anisotropic growth (of the IRS and of the cortex) explains that the spine furrow is eventually located anteriorly rather than posteriorly, in contrast with the orientation of the DP’s curvature.

### Computer simulations

We simulated deformation of the various compartments of the *Acomys* spine follicle under both growth of the IRS and keratinization of the medulla and cortex (see Additional file 1: Figure S7; Additional file 2: Movie S2; Additional file 1: Text; and Appendices I–IV for details). We modeled the posterior and anterior cortex, the medulla, and the IRS (Huxley’s layer, in particular) as distinct solid materials that exhibit linear elasticity. Using the finite-element method implemented in COMSOL Multiphysics v4.3, we simulated the three main processes that shape the *Acomys* spine: (i) the stress applied to the anterior cortex by the growth of the anterior IRS, (ii) the strain exerted to the cortex cells as they elongate (parallel to the long axis of the spine) during the keratinization process, and (iii) the collapse (i.e., sharp decrease of the Young’s modulus, a stiffness measure of elastic materials) of the foam-like keratinized medulla. Several parameters of the model were estimated from biological data: the position and shape distribution of the IRS protrusion, as well as the tempo of medulla cells keratinization were defined on the basis of HE-stained serial sections; the maximum strain of the keratinized cortex cells was measured by the deformation of nuclei on longitudinal sections; and the density of the *Acomys* medulla (modeled as a closed-cell foam) was assumed to be the same as that found in porcupine quills. Other model parameters were optimized to fit experimental observations. Note that the simulation results are robust to variation of the cortex Poisson’s ratio (i.e., the negative ratio of transverse to axial strain). Implementation of these three phenomena supported their prime role in shaping the spine follicle as they were sufficient to replicate the time evolution of the shapes and relative transverse-section areas of the *Acomys* spine layers (Figure 3C–G, Additional file 1: Figure S7, Additional file 2: Movie S2). Additional simulations, removing one process at a time, indicate that it is the combination of all three events listed above that gives rise to the particular shape of the *Acomys* spine.

### The shape and size of the DP correlates with the shape and size of the follicle

We suggest that the crescent shape of the DP and its off-center position (i.e., it is shifted to the anterior side) not only maintain the difference of sizes between anterior and posterior matrix but also trigger the growth of the anterior IRS. Indeed, measurements of minimal distance of proliferating cells from the DP indicate that the mean range of DP signaling reaches the anterior (but not the posterior) IRS, probably explaining the rapid increase in the size of the anterior IRS (Figure 3C–F, Figure 4B, Additional file 1: Figure S4D) and the number of cells (and BrdU^+^ cells) it is made of (Additional file 1: Table S2).

Both in humans [28] and mice [29], it has been suggested that the number of cells in the DP directly correlates with the number of medulla cells in transverse section. In mice, approximately 50 cells make up the DP and the corresponding awl hair exhibit 2 to 3 medulla cells in transverse sections [29]. Our measurements in *Acomys* (Additional file 1: Table S3) give similar ratios, as we count an average of 1,093 cells in the spine DP, associated with a mean of 80 medulla cells in transverse section (corresponding to the follicle level shown in Figure 3E). When moving along the long axis of the follicle towards the skin surface, we additionally observe a correlation between the decrease in the number of cells in the DP and the decrease in the number of BrdU^+^ cells in both the anterior and posterior cortex (Additional file 1: Figure S8).

Longitudinal sections of mouse awl hairs (Additional file 1: Figure S1) indicate a flame-shaped DP, whose transverse section is circular or elliptic/oblong but never crescent shaped. Using BrdU labeling (Additional file 1: Figure S1E) and pH3 staining (Additional file 1: Figure S1F), we and others [30] could not evidence asymmetric proliferation of the IRS cells division around the DP of the laboratory mouse awl hairs but our Keratin 71 staining nevertheless reveals a slight asymmetry of the IRS (Additional file 1: Figure S1G).

### *Spine morphogenesis in* Acomys

**Figure 5:**
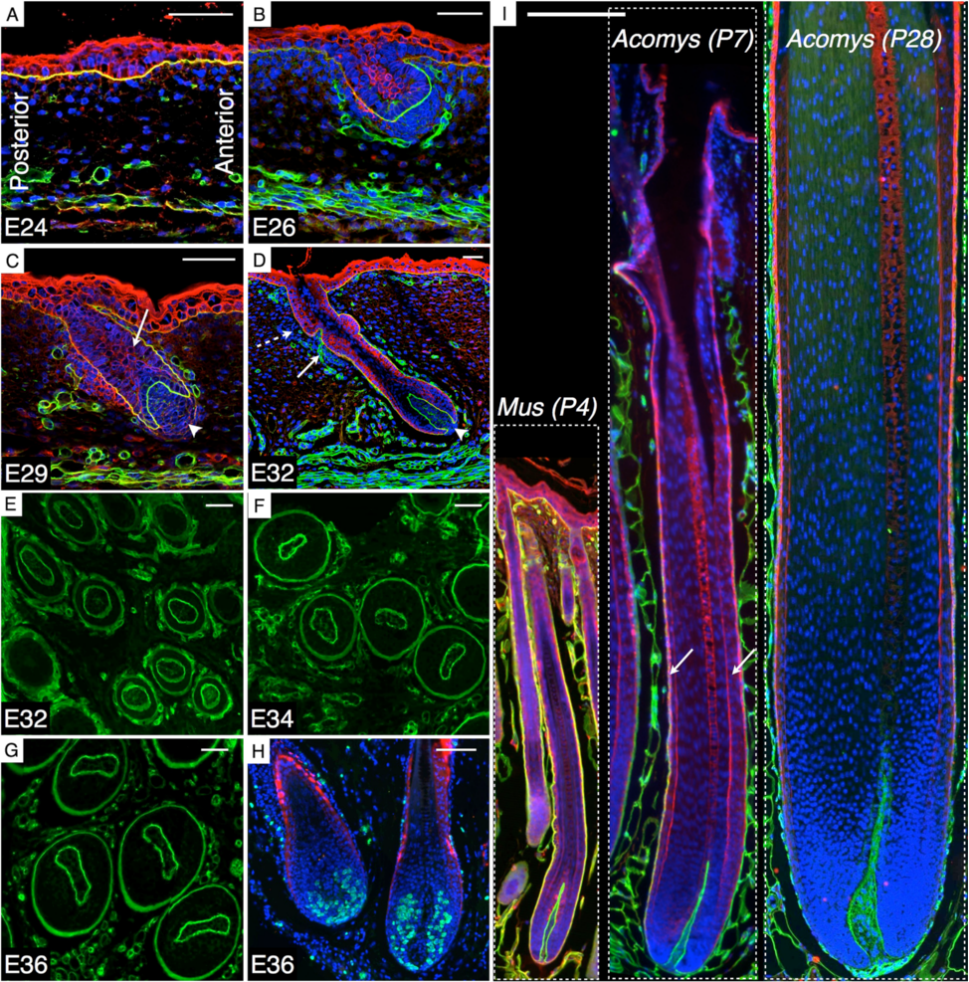
***Acomys *spine morphogenesis.** Pan-Keratin (red) and Laminin (green) immunostaining of lower back embryonic skin at the placode stage at E24 **(A)**, the hair peg stage at E26 **(B)** and at E29 **(C**, arrow: IRS cone), and the bulbous peg stage at E32 **(D**, arrow: bulge, dashed arrow: sebaceous gland). **(E–G)** Laminin-stained transverse sections of E32, E34, and E36 embryonic skin showing the evolving shape of the DP. **(H)** Longitudinal section of two follicles at E36 stained with BrdU and K71. Scale bars: 50 μm. **(I)** Anagen hair on the lower back of *Mus musculus* (P4) and spine on the lower back of *Acomys dimidiatus* (P7 and P28); arrows: IRS. Scale bar: 200 μm.

To further understand the developmental basis of the asymmetry and the increased size of the *Acomys* spines, we performed immunohistochemistry on longitudinal and transverse sections of embryonic samples at different developmental stages. Similarly to the development of epidermal thickenings (placodes) at E14 in the laboratory mouse [31], we observe initiation of hair follicle morphogenesis in *Acomys* (at E24) with the formation of placodes (Figure 5A). The placode cells then divide and grow downwards through the dermis to form a hair peg at about E26 (Figure 5B). These cells eventually surround a dermal condensation that gives rise to the DP (Figure 5C, arrowhead), while the IRS cone forms (Figure 5C, arrow). As the follicle reaches the bulbous peg stage (E32), the bulge, a niche of stem cells, and the sebaceous glands both become visible (Figure 5D). At the following stages, the follicle continues its downward growth and the asymmetry is initiated as the shape of the DP changes from circular to oblong and then to crescent, and its position shifts from the center of the follicle to its anterior side (Figure 5E–G). Cell proliferation is detected at the anterior and posterior side of the follicle (Figure 5H). Then, the distinct layers of the *Acomys* large follicle differentiate, the hair canal opens, and the hair shaft reaches the skin surface (Figure 5I, central panel). The second cycle of hair growth (first postnatal cycle) starts around 30 days after birth [14] and generates even larger follicles (Figure 5I, right panel) producing spines with a distinctive orange band, transforming the juvenile grey pelage into the adult orange spiny fur.

Placodes are larger for *Acomys* than the laboratory mouse and, at the peg stage, follicles are longer and their width is double than in the laboratory mouse (mouse measurements based on Figure 2 from [31]). During the transition to the bulbous peg stage, the laboratory mouse follicle length doubles, whereas, in *Acomys*, it quadruples and its width increases by 20%. At birth, the skin-embedded part of the *Acomys* follicle exhibits a diameter at least double that of the laboratory mouse follicle, and an adult spine is yet double the size of a juvenile *Acomys* hair (Figure 5I). Thus, the spine follicle is not simply a normal-sized follicle that then grows bigger than its *Mus* counterpart, but it is enlarged from the onset of its formation (i.e., the placode stage) and rapidly becomes asymmetrical.

## Discussion

Here, we focused on the development of the spines of the spiny mouse, *Acomys dimidiatus*. We identify the *Acomys* spine as a highly-derived homolog of the laboratory mouse awl hair and show how its morphogenesis brings about its increased size and asymmetrical morphology. Our analyses demonstrate that the *Acomys* awl hair follicles originate from enlarged placodes that are characterized by a rapid downwards growth which results in very large hair shafts. In the process, the DP adopts a crescent shape, directly influencing the whole follicle development: the medulla retains the DP crescent shape, the asymmetrical matrix produces an asymmetrical cortex, and the anterior IRS greatly enlarges, inducing the formation of a deep furrow on the spine shaft.

The spatial distribution of BrdU^+^ cells in the matrix suggests that proliferation is controlled by signaling from the DP. Indeed, whereas the entire thickness of the anterior matrix includes BrdU^+^ cells, the portion of the posterior matrix most distant from the DP is void of BrdU^+^ cells. This distribution is likely to reflect the range of action (dotted line, Figure 3B,C) of growth signals that the DP sends to the surrounding matrix. This hypothesis will need to be tested in the growing *Acomys* follicle through analyses of expression patterns of genes involved in signal transduction pathways (such as Wnts and BMPs) associated with the proliferation of matrix cells [32-34]. When moving up the follicle, the strong correlation between the decrease of cell proliferation and the DP-medulla transition further supports that matrix cell proliferation and cortex differentiation are controlled by the presence and absence of signaling from the DP, respectively. Previous studies on human and mouse hair have already highlighted the importance of the DP in determining follicle size. Our analyses of *Acomys* spines further indicate an impact of the DP shape on that of the whole follicle, making the spiny mouse an ideal model to study such correlations. The role of planar cell polarity genes in the alignment of mouse hair follicles in the skin has been demonstrated [30], but it remains to be seen if the same pathways are involved in the peculiarly shaped DP of the *Acomys* spines. Axial patterning markers, such as Sonic Hedgehog, Krox20, and Igfbp5 [35], could also provide insights on the molecular mechanisms that generate the observed asymmetry.

## Conclusions

Our analyses reveal how hair follicle morphogenesis has been altered during the evolution of the *Acomys* lineage, resulting in a shift from ancestral awl follicles to enlarged asymmetrical spines. Despite the larger difficulties to work with *Acomys* than with laboratory mice, our results provide additional objective reasons to promote *Acomys* as a new model species in mammals as it combines derived skin appendages (this study), spectacular regeneration abilities [8], and medically-relevant physiologies [9,10]. Our study contributes to a better understanding of the evolutionary developmental mechanisms that generated the great diversity of skin appendage phenotypes observed in mammals.

## Abbreviations

3D: Tridimensional; BrdU: 5-bromo-2'-deoxyuridine; DP: Dermal papilla; HEA: Hematoxylin eosin and alcian blue staining; IF: Intermediate filaments; IRS: Inner root sheath; K71: Keratin 71; KPan: Pan-keratin; ORS: Outer root sheath; pH3: Phospho-histone H3.

## Competing interests

The authors declare that they have no competing interests.

## Authors’ contributions

MCM and ACT conceived and supervised the whole study; SAM and ACT performed all experiments; AFM and BC performed numerical simulations; MCM, ACT, and SAM wrote the manuscript. All authors commented on the manuscript and approved the final version.

## Supplementary Material

Additional file 1 Supplementary file including: supplementary text, supplementary bibliography, Appendices I–IV, Supplementary Tables 1–3, Supplementary Figures 1–8, legends of Supplementary movies. Click here for file

Additional file 2 Supplementary Movies 1–2. Click here for file
