# Supplementary material for: Two waves of anisotropic growth generate enlarged follicles in the spiny mouse
Source: EvoDevo. 2014 Sep 25;5:33. doi: 10.1186/2041-9139-5-33 (PMC4335386; doi:10.1186/2041-9139-5-33)
Supplement: Additional file 1 — Supplementary file including: supplementary text, supplementary bibliography, Appendices I–IV, Supplementary Tables 1–3, Supplementary Figures 1–8, legends of Supplementary movies. [file 2041-9139-5-33-S1.pdf]

Two Waves of Anisotropic Growth Generate Enlarged Follicles in the Spiny Mouse

SUPPLEMENTARY MATERIALS

Sophie A. Montandon[1#], Athanasia C. Tzika[1#*], António F. Martins[1], Bastien Chopard[2] & Michel C. Milinkovitch[1*]

1. *Laboratory of Artificial & Natural Evolution (LANE), Dept. of Genetics & Evolution, University of Geneva, Geneva, Switzerland.*
2. *Scientific and Parallel Computing Group, Dept. of Computer Science, University of Geneva, Geneva, Switzerland.*

[#]*these authors contributed equally.*

*Corresponding authors: Michel C. Milinkovitch & Athanasia Tzika

Laboratory of Artificial & Natural Evolution (LANE),

Dept. of Genetics & Evolution, University of Geneva,

Sciences III, 30, Quai Ernest-Ansermet, 1211 Genève 4, Switzerland.

Tel: +41(0)22 379 67 85; Fax: +41(0)22 379 67 95

URL: www.lanevol.org

E-mail: michel.milinkovitch@unige.ch ; athanasia.tzika@unige.ch

Other authors e-mails: sophie.Montandon@unige.ch , antonio.martins@unige.ch , bastien.chopard@unige.ch

Running title (50 characters): Development of *Acomys* spines

Keywords: *Acomys*, spiny mouse, evolutionary developmental biology, hair, spines.

Supplementary text: ***Simulated deformation of the*** **Acomys** ***spine***

We modeled the spine as a cylinder comprised of three layers, the inner root sheath (IRS), the cortex, and the off-centered medulla (Sup. Fig. 7A). We assume that each layer of the follicle is made of a continuous material and with properties inferred from its overall behavior rather than its distinct cellular components. We chose the equation system of solid linear elasticity (cf. Appendix I) as appropriate to describe the characteristics of each layer and the deformations they are subjected to. Three processes were incorporated in the model (Sup. Fig. 7D): *(i)* the protrusion of the IRS within the anterior cortex, *(ii)* the massive keratinization of the cortex, which results in the elongation of its cells following the long axis of the spine, and *(iii)* the apoptosis of the medulla cells that produces a foam-like structure. The main experimental input was serial transverse sections of a follicle stained with Hematoxylin and Eosin. From these, we were able to *(i)* measure the relative transverse-section area of each layer and *(ii)* evaluate the degree of keratinization of the cortex at different levels of the follicle. In addition, we estimated the rate of apoptosis within the medulla by measuring the increase of the surface void of cells. In all cases, a sigmoid function was employed to mirror the gradual transition from one state to the next, as no abrupt modification was observed within the sectioned follicle. The simulations were performed using the finite-element method implemented into COMSOL Multiphysics v4.3.

Simulations start at a height in the spine follicle where the dermal papilla has already acquired its crescent shape and is surrounded by the undifferentiated matrix. In our model, we assume that the transition from matrix to cortex and from dermal papilla to medulla does not entail any alteration in the physical properties of these layers, contrary to the pronounced impact of their keratinization. The first phenomenon considered is the IRS protrusion, which is the result of cellular growth and division. Measuring the stress that this phenomenon exerts on the anterior cortex is not possible, but HE staining indicates that the IRS protrusion *(i)* is symmetrical across the anterior-posterior (AP) axis of the follicle and *(ii)* decreases in intensity as the distance to this axis increases. Thus, we model the protrusion by imposing a stress at the boundary between the anterior IRS and the anterior cortex, with an anterior-to-posterior direction and an intensity distribution following a Gaussian centered on the AP axis (Sup. Fig. 7A, Appendix II). Note that we assume the IRS strain to be constrained outwardly by the outer root sheath (ORS). The follicular height at which this process initiates and the position at which it stabilizes were defined based on anterior IRS area growth measurements performed on serial sections.

The keratinization of cortex cells causes them to elongate parallel to the long axis (z-axis) of the follicle (Sup. Fig. 7B). Furthermore, the stretching of cells in this direction results in the reduction of their transverse section (thinning). Within the framework of deformation theory, this means that the deformed material exhibits a non-zero Poisson's ratio. As such, modeling this step amounts to imposing a strain along the z axis (Appendix III). Even though HE staining of serial sections is sufficient to define the height at which cortex keratinization initiates and completes (Sup. Fig. 7D), it does not provide information on the amount of thinning of the cells. Given the correlation between cellular and nuclear shapes [1], we estimated the magnitude of elongation of the keratinized cortex cells by measuring nuclei deformation on longitudinal sections stained with

Hoechst (Sup. Fig. 7C). Note that both HE and TUNEL staining reveal a delay in the keratinization of the anterior relative to the posterior cortex (Fig. 2 and 4), a delay that was implemented in our simulations (Sup. Fig. 7D).

In stark contrast with the cortex, medulla cells exhibit little or no z-directional straining during keratinization. However, as they undergo apoptosis, they will leave behind empty cavities delineated by cortex protrusions, forming a closed-cell foam structure, as evidenced by the reticulated material observed under Scanning Electron Microscopy (SEM) (Sup. Fig. 5). We thus modeled the medulla following the equation system proposed by Gibson and Ashby [2] for closed-cell foams (Appendix IV). We estimated the tempo of medulla cells apoptosis by measuring the relative area of 'empty' medulla space in transverse sections (Sup. Fig. 7D). The medulla relative density is assumed to be the same as that found in porcupine quills [3].

In short, the three major processes implemented in our simulations are: *(i)* a stress applied to the anterior cortex because of the IRS protrusion, *(ii)* a vertical strain exerted on the cortex cells as they elongate during the keratinization process, and *(iii)* the formation of a foam-like structure in the keratinized medulla. These three events are sufficient to replicate the evolution of the relative transverse-section areas of the follicle layers and the shape of the concave *Acomys* spine (Sup. Fig. 7E, Sup. movie 2, upper left). The model also allowed us to investigate alternative outcomes, assuming the absence of one of the three processes at a time: IRS protrusion, cortex keratinization, and medulla keratinization (Sup. Movie 2). Even though each process significantly influences the outcome, it is the combination of all that gives rise to the particular concave spine we observe.

Supplementary Bibliography

1. Versaevel M*, et al*: **Spatial coordination between cell and nuclear shape within micropatterned endothelial cells.** *Nature communications* 2012, **3:**671.
2. Gibson LJ, Ashby MF: *Cellular Solids Structure and Properties*. Cambridge, UK: Cambridge University Press; 1999.
3. McKittrick J*, et al*: **The Structure, Functions, and Mechanical Properties of Keratin.** *Jom-Us* 2012, **64:**449-468.

Appendix I: Linear elasticity equations

The field equations of linear elasticity take on the general form

$$\nabla.\sigma + F = \rho\ddot{u}\,, \qquad \text{(equation 1)}$$

$$\sigma = C : \varepsilon\,, \qquad \text{(equation 2)}$$

$$\varepsilon = \frac{1}{2}\left[\nabla u + (\nabla u)^T\right], \qquad \text{(equation 3)}$$

where σ is the stress tensor, F is the body force per unit volume, ρ is the density, u is the displacement field, C is the fourth-order stiffness tensor, and ε is the strain tensor. In addition, ∇ represents the nabla operator, *:* stands for tensorial product, and $\ddot{u}$ is the second-order time derivative of the displacement field.

In the case of follicle development, several simplifications may be considered:

- ✓ There are no body forces, *i.e.*, the effects of long-range interactions (such as gravity) can be neglected;
- ✓ The system is always in a quasi-static regime, *i.e.*, one may neglect the propagation of elastic waves;
- ✓ Each follicle compartment can be considered as an isotropic material (but see notes below on the simulation of the IRS). Unlike the previous two, this assumption is less obvious as cells may indeed exhibit anisotropic properties, in particular those inside the cortex region during and after straining. Nevertheless, given the lack of *a priori* knowledge on the effect, it seems reasonable to assume isotropy as a starting point.

Under these assumptions, the first equation simplifies to:

$$\nabla.\sigma = 0 \qquad \text{(equation 4)}$$

and the stiffness tensor is fully specified by the bulk modulus K and the shear modulus G according to

$$C_{ijkl} = K\delta_{ij}\delta_{kl} + G\left(\delta_{ik}\delta_{jl} + \delta_{il}\delta_{jk} - \frac{2}{3}\delta_{ij}\delta_{kl}\right) \qquad \text{(equation 5)}$$

where δ_{ij} is the Kronecker delta symbol. This allows one to write straightforward linear relations between stress and strain:

$$\sigma_{ij} = K\delta_{ij}\varepsilon_{kk} + 2G\left(\varepsilon_{ij} - \frac{1}{3}\delta_{ij}\varepsilon_{kk}\right) \qquad \text{(equation 6)}$$

$$\varepsilon_{ij} = \frac{1}{9K}\delta_{ij}\sigma_{kk} + \frac{1}{2G}\left(\sigma_{ij} - \frac{1}{3}\delta_{ij}\sigma_{kk}\right) \qquad \text{(equation 7)}$$

where summation over repeated indices is implied. Note that each material (*i.e.*, each follicle compartment) has its own values for K and G.

Appendix II: IRS protrusion

Mathematically, the stress applied by the IRS to the anterior cortex equates to a boundary stress of the form

$$|\sigma_{IRS}| = \sigma_{\max} \exp\left[-\left(\frac{d_{AP}}{S_{AP}}\right)^2\right] / \left[1 + \exp\left[-k_{IRS}(z - z_{IRS})\right]\right] \qquad \text{(equation 8)}$$

where the stress is always applied on the anterior-to-posterior direction, σ_{max} is the maximum stress applied by the IRS on the cortex, d_{AP} is the distance to the AP axis of the follicle, S_{AP} is a measure of the spatial dispersion of the stress profile along the IRS/cortex boundary, and Z_{IRS} and K_{IRS} control the position of the inflection point and the rate (*i.e.*, derivative at the inflection point) of the sigmoid describing the effect along the z axis (Sup. Fig. 7). Using measurements on HE-stained serial sections and considering z=0 at the base of the simulated follicle, we estimated the values Z_{IRS}=10 and K_{IRS}=0.3. The maximum stress σ_{max} cannot be objectively estimated from observations, but its value may be set to σ_{max} =1 by performing a dimensionalization of the stress variables and constants, *i.e.*, measuring all the stress components and elastic parameters *K*, *G* in units of σ_{max}. The dispersion constant S_{AP} is not directly measured, but we can reasonably assume that its value lies in the range 50-100 µm (on the basis of measurements on the actual follicles), and thus we set it to S_{AP}=80 µm. We set the IRS's Poisson's ratio to 0.45 and its Young modulus (*E*) to be dependent on the state of stress: when subject to tension it has a low modulus of $1.25E^{-5}$ (*i.e.*, it offers little or no resistance to expansion), while increasing sigmoidally to a higher value of 0.625 when compressed (to model its resilience when "squeezed" by the components of the hair shaft). This allowed us to simulate the process with IRS strain as equivalent of IRS growth.

Appendix III: Cortex keratinization

The phenomenon may be simulated by subjecting the cortex to a strain given by

$$\varepsilon_{33}(z) = \varepsilon_{\max} / \left[1 + \exp\left[-k_{cortex}(z - z_{cortex})\right]\right] \qquad \text{(equation 9)}$$

where ε_{33} is a strain in the z-direction, ε_{max} is the maximum strain, while z_{cortex} and k_{cortex} are the position and derivative of the sigmoid inflection point, respectively: $z_{cortex,posterior}$=85, $z_{cortex,anterior}$=95, k_{cortex}=0.08; all expressed in units corresponding to the section number. It should be noted that z_{cortex} differs between the anterior and posterior sides to account for a slight phase difference between the two. The maximum strain ε_{max} was estimated to ≈0.62 from the nuclear strain visible in Hoechst stained longitudinal sections.

The z-directional stress may be expressed in terms of the z-directional strain according to

$$\sigma_{33} = E\varepsilon_{33} + \nu(\sigma_{11} + \sigma_{22}) \qquad \text{(equation 10)}$$

Plugging this expression onto

$$\varepsilon_A = \varepsilon_{11} + \varepsilon_{22} \qquad \text{(equation 11)}$$

one can obtain the result

$$\varepsilon_A = -2\nu\varepsilon_{33} + \frac{(1+\nu)(1-2\nu)}{E}(\sigma_{11} + \sigma_{22}) \qquad \text{(equation 12)}$$

where ε_A is the area strain (a measure of its relative reduction of cross-section area). Equation 12 indicates that the z-directional strain and the planar cross-sectional strains will dominate the system for high and low Poisson's ratios, respectively. Although this result does not allow one to fully constrain the parameter (due to the influence of the second term), it can be used to narrow considerably its region of accepted values: too low a number would produce insufficient decrease, while too high a value would cause too much shrinkage. Within our model, we find that $\nu \approx 0.3$-0.475 robustly fit the experimental observations and we chose the value $\nu=0.425$ (Sup. Fig. 7E) for both cortexes. The Young modulus of the cortex is set to $E=1.25$.

Appendix IV: Medulla keratinization

Following Gibson and Ashby [2], the elastic modulus of a closed-cell foam structure can be calculated using the relative density of the foam and the substance composing its walls ($R_\rho = \rho_{foam} / \rho_{wall}$), the fraction of solid contained in the foam's cell edges (Φ), and the elastic modulus of the wall material E_{wall}, according to

$$E_{foam} = E_{wall}\left[\phi^2 R_\rho^2 + (1-\phi) R_\rho\right] \qquad \text{(equation 13)}$$

In the particular case of the follicle's development, the 'wall' substance will mostly be the cortex's keratinized protrusions. Assuming once more a gradual (sigmoid) process, we model the medulla's apoptosis and consequent transition to a foamy structure by modifying its elastic modulus (in agreement with equation 13) as follows:

$$E_{med}(z) = E_{med,ini} + \Delta E / \left[1 + \exp\left[-k_{medulla}(z - z_{medulla})\right]\right]$$

$$\Delta E = -E_{med,ini} + E_{cortex}\left[\phi^2 R_\rho^2 + (1-\phi) R_\rho\right] \qquad \text{(equation 14)}$$

where $E_{med,ini}$ is the initial (before apoptosis) Young modulus (set to $E_{med,ini}=1.25$) of the medulla and $\Delta E + E_{med,ini}$ is the Young modulus of the foam made up of cortex protrusions. Note that E can easily be calculated from the values of K and G using the relation

$$E = \frac{9KG}{3K+G} \qquad \text{(equation 15)}$$

As in the other processes (Appendices I-III), the constants $z_{medulla}$ and $k_{medulla}$ characterize the shape of the sigmoid and were set to: $z_{medulla}=115$, $k_{medula}=0.3$.

Given the almost constant cross-sectional area and the absence of straining in the z-direction, we constrain the Poisson's ratio to be very close to 0.5 ($\nu=0.4995$), the theoretical limit of incompressibility. This can also be seen from the formula for ε_A given in Appendix III: if $\varepsilon_{33}=0$ and $\nu=0.5$, then $\varepsilon_A=0$.

Estimating the parameter Φ for the case at hand is essentially unattainable. Nevertheless, a large number of closed-cell foams exhibit values in the very narrow range of 0.6-0.8 [2]. We made the same assumption for the development of the *Acomys* follicle and set $\Phi=0.8$.

The relative density of the medulla foam R_ρ was assumed to be the same as that found in porcupine quills [3], *i.e.*, $R_\rho \approx 0.1$.

Supplementary Table S1. ***Absolute (in µm²) and relative (% of total) transverse-section areas of the different layers of*** **Acomys** ***spines.*** Measurements were performed on Hematoxyline/Eosin stained serial sections that correspond to levels shown in Figure 3 (but on images of a different follicle). The table shows how the relative areas of the various layers (DP/medulla, IRS, and matrix/cortex) change along the long axis of the follicle, with the posterior cortex (indicated in grey) displaying a remarkable reduction of relative area.

	DP/Medulla		Anterior IRS		Anterior matrix/cortex		Posterior matrix/cortex		Total
	Abs (μm^2)	Rel (%)	Abs (μm^2)	Rel (%)	Abs (μm^2)	Rel (%)	Abs (μm^2)	Rel (%)	Abs (μm^2)
Fig. 3A	15,531	33.3	0	0.0	0	0.0	31,081	66.7	46,612
Fig. 3B	15,017	18.9	3,018	3.8	27,128	34.1	34,407	43.2	79,570
Fig. 3C	22,085	26.8	8,318	10.1	11,631	14.1	40,485	49.1	82,519
Fig. 3D	18,843	23.3	10,093	12.5	13,047	16.1	38,994	48.2	80,977
Fig. 3E	17,682	23.0	11,145	14.5	15,968	20.8	32,060	41.7	76,855
Fig. 3F	18,222	24.8	8,090	11.0	15,941	21.7	31,122	42.4	73,375
Fig. 3G	20,189	35.5	11,621	20.4	9,626	16.9	15,455	27.2	56,891

Supplementary Table S2. ***Cycling cell (BrdU+) densities and total cell densities in* Acomys *follicles*** based on 24 serial sections of two different follicles of a 22-days old *Acomys* (*I* and *II*) and 41 serial sections of two different follicles of a 28-days old *Acomys* (*III* and *IV*). The inset image highlights the different areas in which counts were performed. Values in the two shaded rows indicate that the percentage of cycling cells is similar in the anterior and posterior Matrix, but substantially greater in the anterior than the posterior IRS.

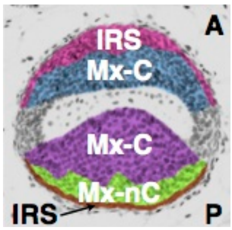

		Posterior				Anterior			
	Follicle number ➔	*I*	*II*	*III*	*IV*	*I*	*II*	*III*	*IV*
**Mx-C = Matrix Cycling (BrdU+) area**	Area (mm^2)	0.17	0.13	0.38	0.40	0.15	0.16	0.34	0.31
	BrdU+ cells	1,412	988	2,703	2,842	1,359	1,408	2,934	2,483
	Total cells (Hoechst)	3,191	2,040	5,961	6,028	3,261	3,172	6,557	6,017
	% cycling cells	44.25	48.43	45.34	47.15	41.67	44.39	44.75	41.27
	Cell density (cells/mm^2)	18,422	16,075	15,687	15,144	21,503	20,453	19,285	19,497
**Mx-nC = Matrix Non-Cycling area**	Area (mm^2)	0.38	0.54	0.84	0.91	0.08	0.21	0.45	0.45
	BrdU+ cells	49	64	45	30	45	28	46	37
	Total cells (Hoechst)	4,342	5,895	9,971	10,719	1,520	3,446	7,062	7,999
	% cycling cells	1.13	1.09	0.45	0.28	2.96	0.81	0.65	0.46
	Cell density (cells/mm^2)	11,528	10,996	11,870	11,833	18,140	16,445	15,693	17,933
**IRS**	Area (mm^2)	/	/	0.13	0.15	/	/	0.24	0.24
	BrdU+ cells	/	/	45	40	/	/	233	196
	Total cells (Hoechst)	/	/	2,080	2,194	/	/	3,948	4,453
	% cycling cells	/	/	2.16	1.82	/	/	5.90	4.40
	Cell density (cells/mm^2)	/	/	15,614	14,936	/	/	16,096	18,772
**Total area**	Area (mm^2)	0.55	0.66	1.22	1.30	0.24	0.36	0.79	0.75
	BrdU+ cells	1,461	1,052	2,748	2,871	1,404	1,436	2,980	2,520
	Total cells (Hoechst)	7,533	7,935	15,932	16,747	4,781	6,618	13,619	14,016
	% cycling cells	19.39	13.26	17.25	17.14	29.37	21.70	21.88	17.98
	Cell density (cells/mm^2)	13,700	11,968	13,059	12,844	20,306	18,149	17,239	18,573

Supplementary Table S3. ***Dermal papilla and medulla cell counts.*** Numbers of DP cells were counted on successive transverse sections (on average 36) of ten follicles, whereas numbers of medulla cells represents the average count among three sections at a level that corresponds to Fig. 3E. The sections were stained with Pan-Keratin, Laminin and Hoechst. All follicles are from the lower back of a 32-days old *Acomys*. The ratio of DP to medulla cells roughly corresponds with the one found in mouse hair awl, namely follicles with approximately 50 DP cells exhibit 2 to 3 medulla cells. *SD*, standard deviation.

	**No. of DP cells**	**Average no. of medulla cells**
Follicle 1	1,038	80
Follicle 2	1,074	80
Follicle 3	1,067	86
Follicle 4	1,052	87
Follicle 5	1,055	/
Follicle 6	1,161	79
Follicle 7	1,043	77
Follicle 8	1,174	82
Follicle 9	1,113	74
Follicle 10	1,152	77
**Mean ± SD**	**1,093 ± 54**	**80 ± 4**

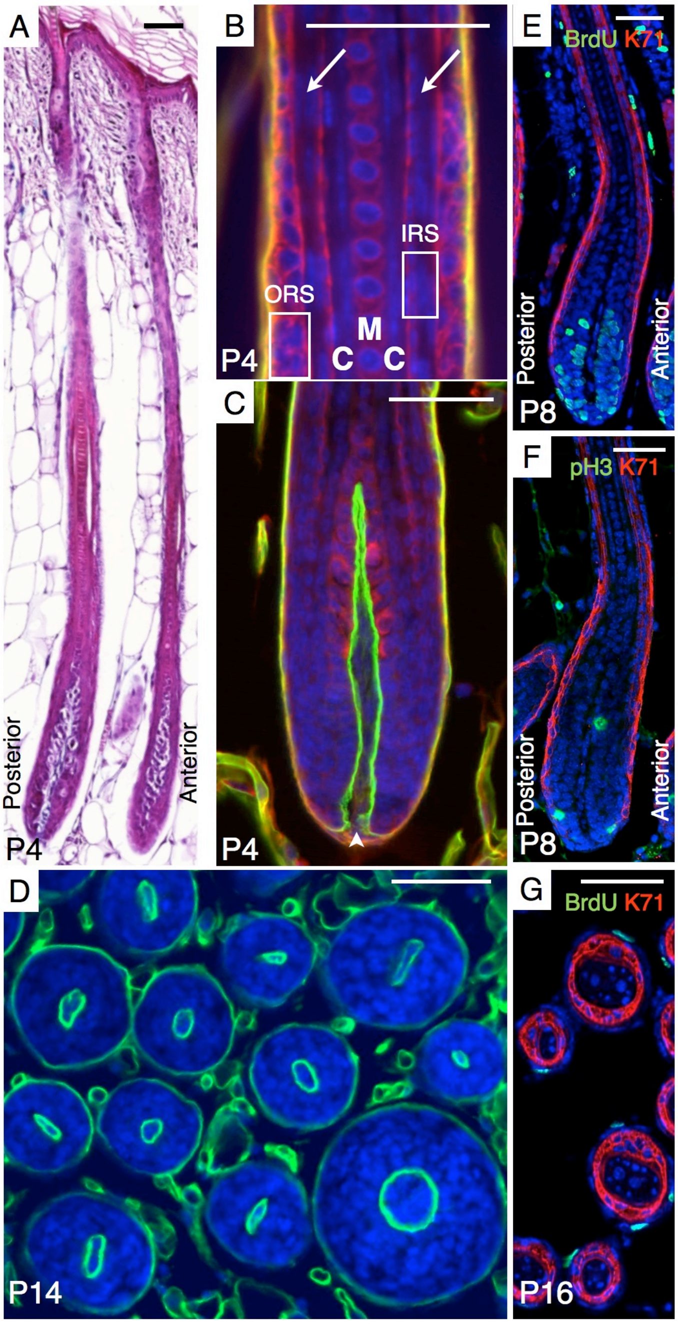

Supplementary Figure S1. ***Laboratory mouse awl hair morphology, first anagen.*** (**A**) Hematoxylin/Eosin (H&E) staining at P4. (**B**) Follicle layers highlighted by the Pan-Keratin antibody: ORS, IRS, cortex (C), and medulla (M). Note the symmetry of the IRS indicated by arrows. (**C**) The laboratory mouse follicle exhibits a symmetrical dermal papilla. (**D**) Transverse section of the dermal papilla (green) can be circular or elongated but never crescent shaped. (**E**) Longitudinal section at P8: BrdU (green), K71 (red) and Hoechst (blue) immunostaining. (**F**) pH3 (green), K71 (red) and Hoechst (blue) immunostaining. (**G**) Transverse section at P16, BrdU (green), K71 (red) and Hoechst (blue) immunostaining. Note that the IRS can be a bit asymmetrical at this stage. Scale bars: 50 µm.

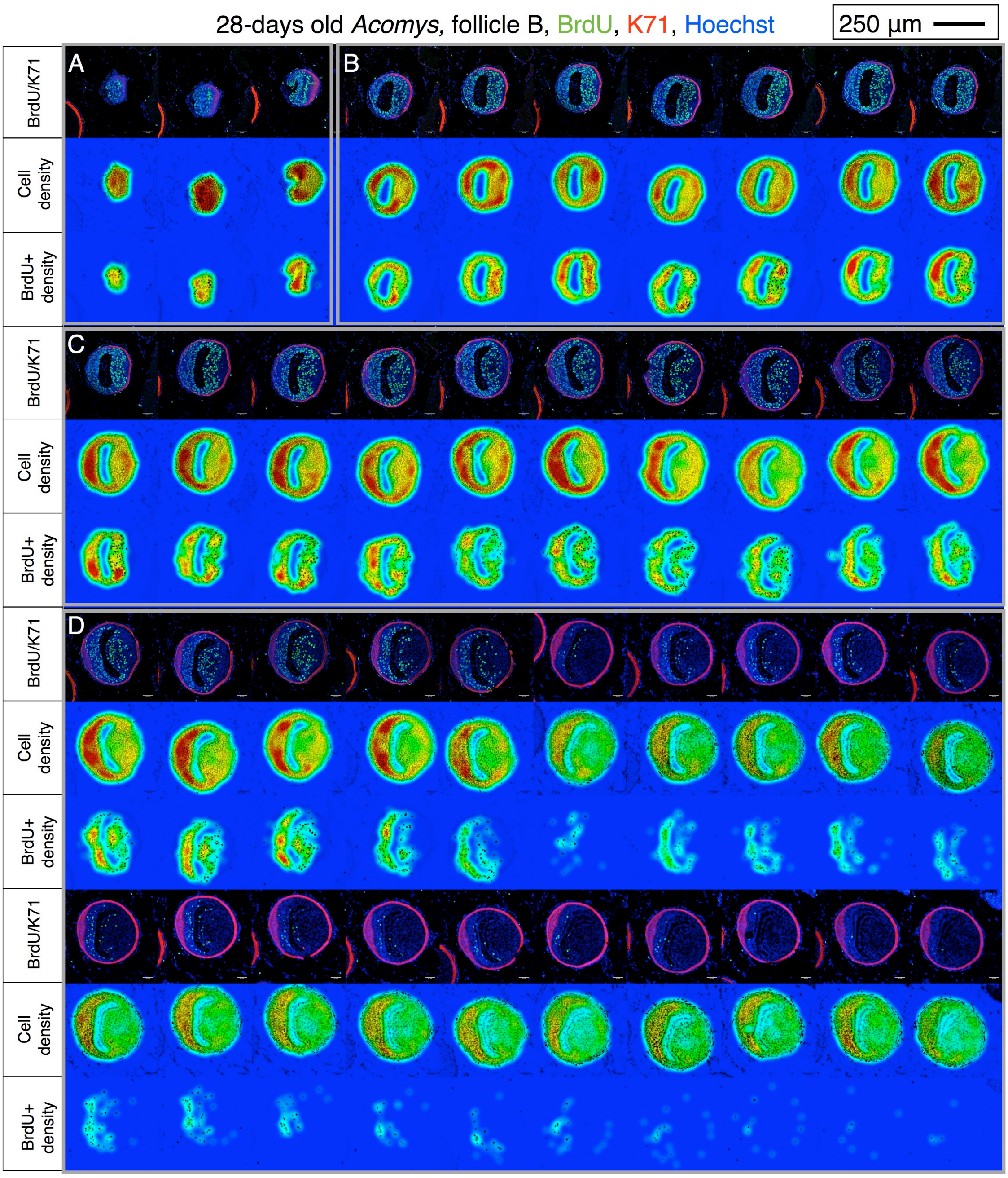

Supplementary Figure S2. ***Cycling cells of an* Acomys *spine follicle.*** BrdU, K71 and Hoechst immunostaining and corresponding color-coded densities (blue: low density, red: high density) for all cells and BrdU+ cells. The color range is arbitrarily and separately defined for total cell density and BrdU+ cell density. The boxes labeled **A**, **B**, **C**, and **D** correspond to the different levels in the follicle as described in the text and presented in Figure 3. Scale bars: 250 µm.

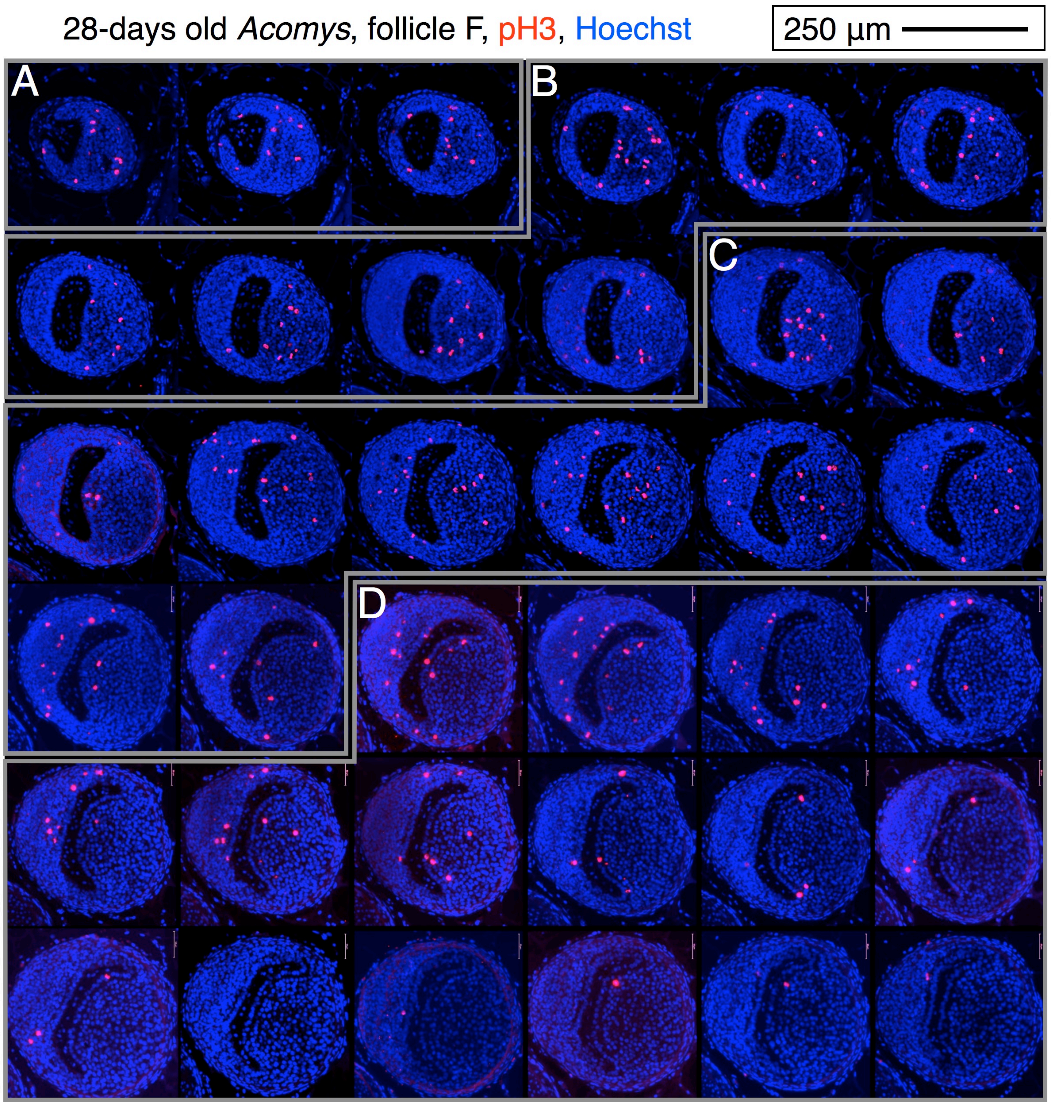

Supplementary Figure S3. ***Dividing cells of a spine follicle.*** Red and blue signals correspond to pH3 and Hoechst immunostaining, respectively. The boxes labeled **A**, **B**, **C**, and **D** correspond to the different levels in the follicle as described in the text and presented in Figure 3. Scale bars: 250 µm.

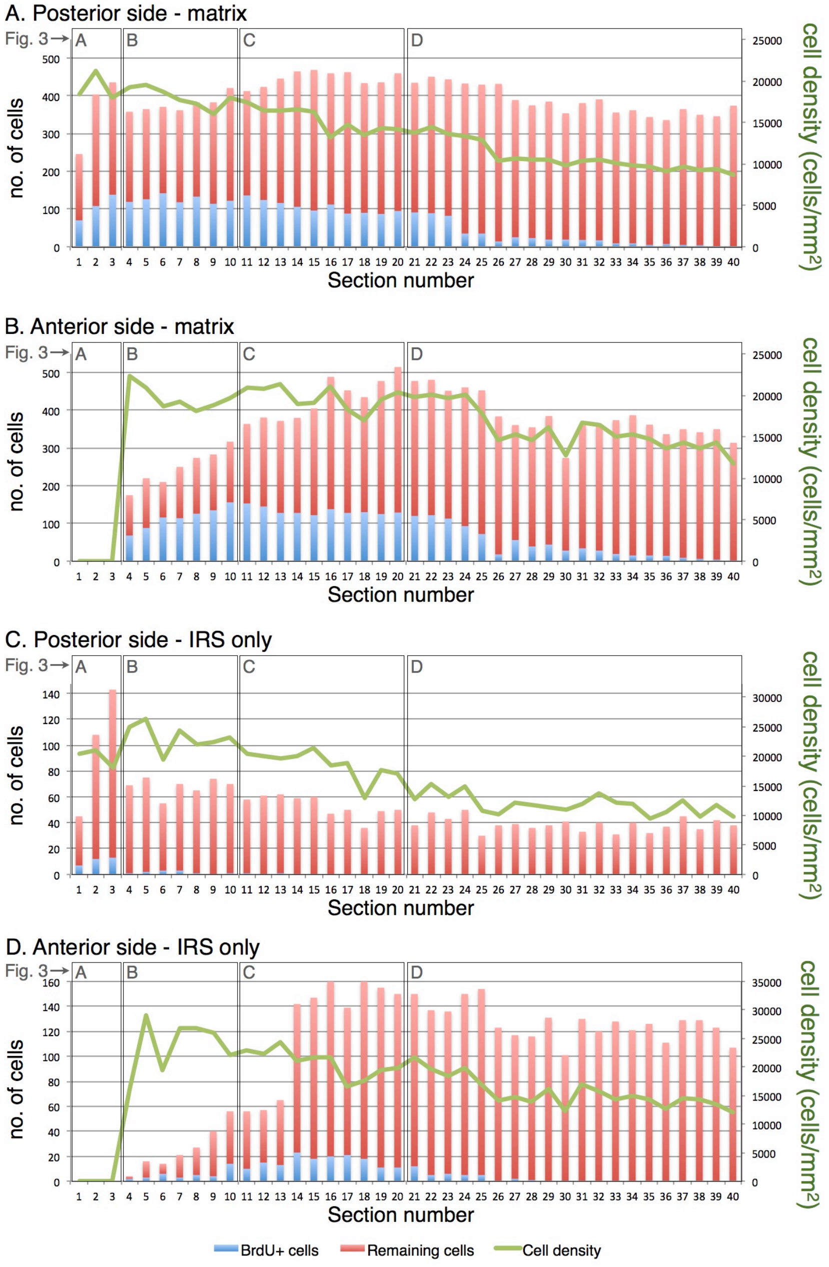

Supplementary Figure S4. ***Number of cycling and non-cycling cells and total cell density of a spine follicle.*** Number of cells were counted on serial transverse sections of a lower back spine from a four weeks-old *Acomys,* at the posterior and anterior sides of the follicle, as well as the posterior and anterior IRS. The blue part of each column represents the cycling cells and the red the remaining ones. The green line indicates the corresponding total cell density. The boxes labeled **A**, **B**, **C**, and **D** correspond to the different levels in the follicle as described in the text and presented in Figure 3.

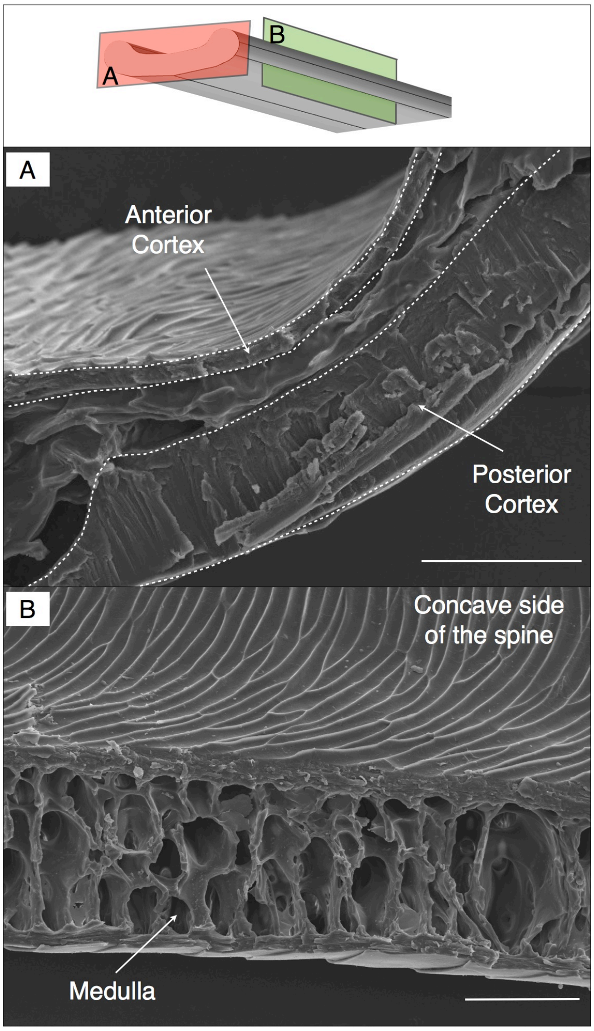

Supplementary Figure S5. ***Medulla and cortex keratinization.*** Scanning electron microscopy images of a fractured spine from the lower back of an adult *Acomys*. (**A**) Transverse section showing the compact form of the cortex, and (**B**) longitudinal section of the medulla presenting its reticulated (foamy) structure. Scale bars: 50 µm.

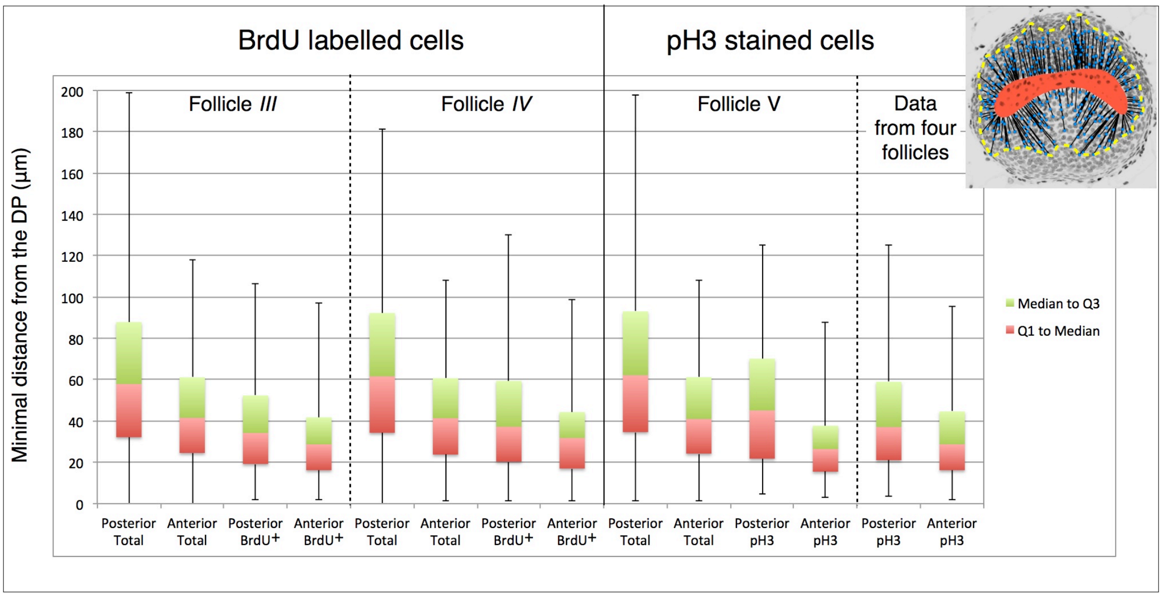

Supplementary Figure S6. ***Minimal distance between the dermal papilla and matrix cells (all cells or proliferating cells only).*** Box-plot indicating total range of distances as well as distances for the first quartile to the median (red) and from the median to the 3rd quartile. The median and range of distance distributions are similar for the anterior and posterior matrix when cycling (BrdU $^{+}$) or dividing (pH3) cells are considered despite that the median and range of distance distribution are substantially larger for the posterior than for the anterior matrix when all cells are considered. The inset image highlights how minimal distances (black lines) of cells (blue dot) from the dermal papilla (red zone) were measured. The yellow line depicts the border of the spatial distribution of cycling cells. Note that that anterior (but not posterior) IRS cells are included in that span.

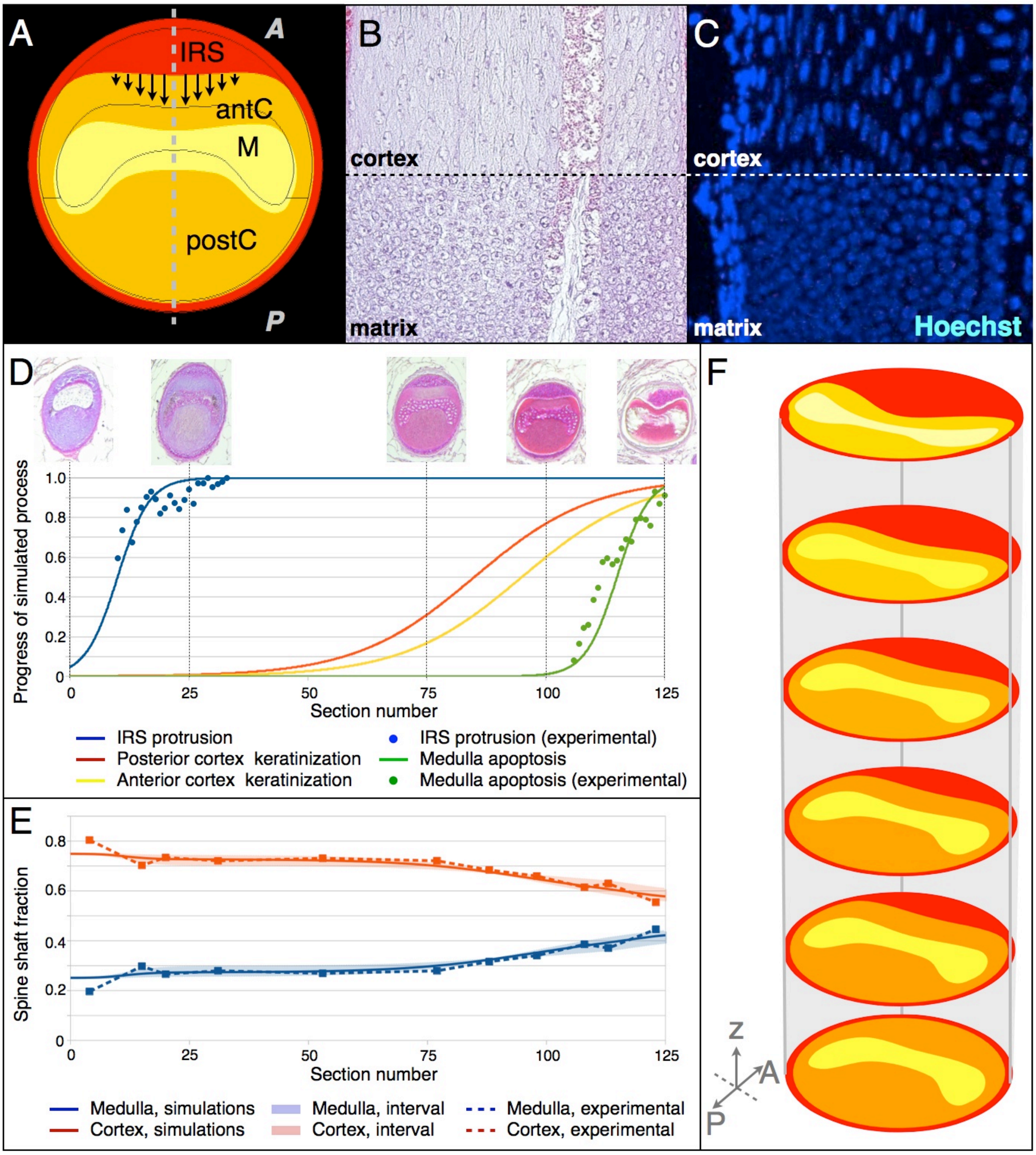

Supplementary Figure S7. ***Simulating the*** **Acomys** ***spine development.*** (**A**) Cross-section of a simulated follicle; dashed line, AP axis; black arrows, stress applied by the anterior IRS on the cortex; *IRS*, inner root sheath; *antC/postC*, anterior and posterior cortex; *M*, medulla. HE (**B**) and Hoechst-stained (**C**) follicles longitudinal sections showing that cortex cells (but not matrix cells) exhibit elongated cells bodies and elongated nuclei along the z-axis of the follicle. (**D**) Simulated dynamics (plain lines) of the three processes (IRS protrusion, cortex cells keratinization, and medulla cells apoptosis) that shape the follicle along the z axis. Dots represent experimental observations: increase of relative transverse-section area of the IRS (blue) and accumulation of cavities with no cells in the medulla (green). (**E**) Relative (% of total) transverse-section areas of the cortex and medulla, as measured on a real follicle (dashed lines; Sup. table 1) and in simulation runs (plain lines). The shaded area represents intervals of simulation results with Poisson's ratio values ranging from 0.3 to 0.475. (**F**) Transverse sections of the simulated *Acomys* follicle along the z axis. *A*: anterior, *P*: posterior.

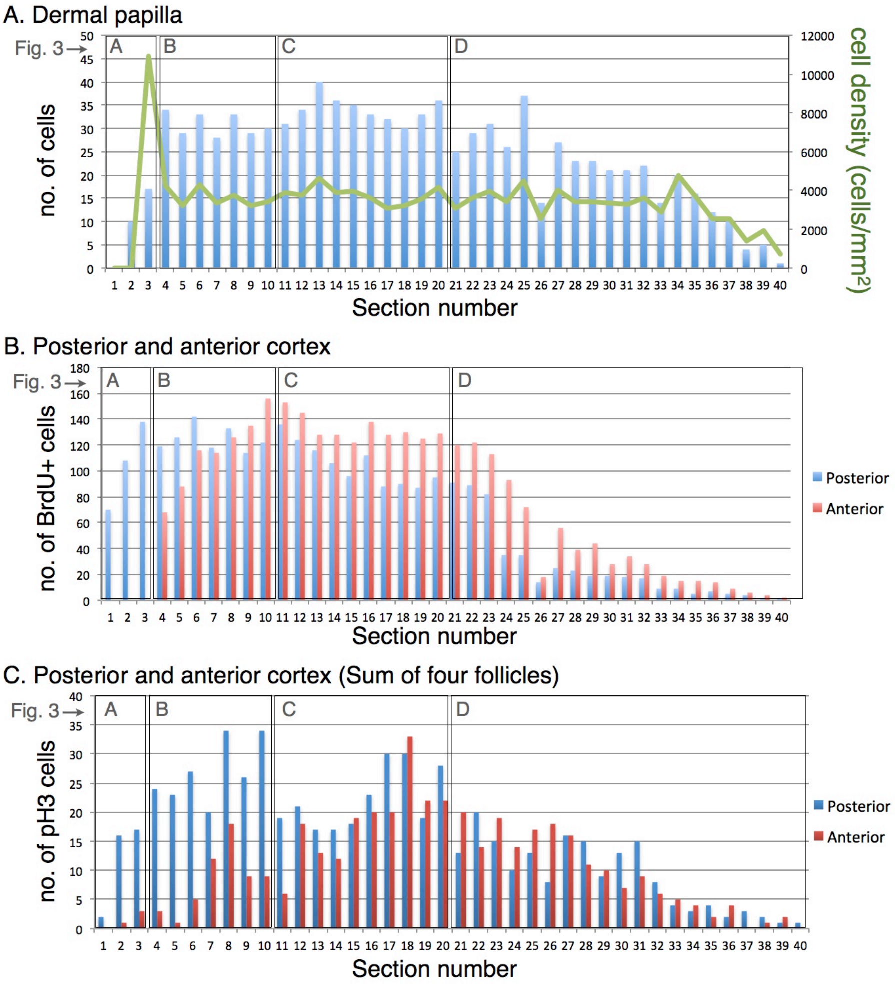

Supplementary Figure S8. ***Cell counts (from transverse sections of a spine follicle) in the dermal papilla and cortex.*** Upper panel: the number of cells per section (columns) and the corresponding cell density (line) are presented for the dermal papilla. For comparison, the middle and lower panels respectively show the number of BrdU-labeled and pH3-stained cells at the posterior (blue) and anterior (red) sides of each section. The boxes labeled **A**, **B**, **C**, and **D** correspond to the different levels in the follicle as described in the text and presented in Figure 3.

Supplementary Movie S1. Acomys ***spine 3D reconstruction.*** 102 transverse sections in a spine follicle were stained using Pan-Keratin and Laminin and aligned. The 3D reconstruction shows that the dermal papilla exhibits a curved geometry (crescent-shape in transverse section) with two distal lobes. The medulla takes the cross-section shape and position of the underlying DP in the resulting hair shaft. The growth of the anterior IRS is also visible.

Supplementary Movie S2. ***Simulated dynamics of the*** **Acomys** ***spine follicle development.*** Upper left panel: movie showing time evolution of virtual transverse sections of a simulated follicle, where we observe the crescent-shape of the dermal papilla, the asymmetric cortex and the impact of the IRS protrusion at the anterior side of the follicle. The three other panels show identical simulation except that one of the three following processes was excluded: IRS protrusion, cortex keratinization, and medulla keratinization. Even though each process significantly influences the outcome, it is the combination of all that gives rise to the particular concave spine morphology.
